# Association Between PNPLA3 Inhibition and Gout: A Drug Target Mendelian Randomization Study

**DOI:** 10.1155/ije/6664846

**Published:** 2025-08-20

**Authors:** Chen Wang, Pei Guo, Xiang Liu, Xingxing Xu, Li Zou, Shi Meng, Qing Guo, Qiang Wen, Chuang Yang

**Affiliations:** ^1^Department of Clinical Pharmacy, The First Affiliated Hospital of Chongqing Medical and Pharmaceutical College, Chongqing 400016, China; ^2^Department of Pharmacy, Children's Hospital of Chongqing Medical University, National Clinical Research Center for Child Health and Disorders, Chongqing Key Laboratory of Pediatric Metabolism and Inflammatory Diseases, Ministry of Education Key Laboratory of Child Development and Disorders, Chongqing, China

**Keywords:** gout, Mendelian randomization, metabolic dysfunction–associated steatotic liver disease, PNPLA3, urate

## Abstract

**Aims:** Patatin-like phospholipase domain-containing protein 3 (PNPLA3) plays a crucial role in metabolic dysfunction–related steatotic liver disease. ARO-PNPLA3 is a therapeutic agent designed to target PNPLA3, but its long-term effects remain uncertain. The objective of this study was to ascertain the impact of PNPLA3 inhibition on the risk of gout through Mendelian randomization.

**Methods:** Mendelian randomization analysis was conducted by choosing single nucleotide polymorphisms (SNPs) in proximity to the PNPLA3 gene, which were significantly associated with the percentage of hepatic fat, to represent PNPLA3 suppression. Nonalcoholic fatty liver disease and hepatic fibrosis served as positive controls, while urate and gout were the outcomes.

**Results:** Genetically predicted PNPLA3 inhibition significantly increased the risk of gout (OR: 1.83, 95% CI: 1.49 to 2.26, *p* = 1.44 × 10^−8^), idiopathic gout (OR: 2.42, 95% CI: 1.60 to 3.65, *p* = 2.81 × 10^−5^) and urate (OR: 1.12, 95% CI: 1.01 to 1.23, *p* = 2.56 × 10^−2^), but not with gout due to impairment of renal function (OR: 1.25, 95% CI: 0.37 to 4.22, *p* = 7.23 × 10^−1^).

**Conclusions:** This study found that PNPLA3 inhibition increased the risk of high urate level and gout. In addition, PNPLA3 inhibition also increased triglyceride (TG) levels, which partially mediate the relationship between PNPLA3 inhibition and gout.

**Trial Registration:** ClinicalTrials.gov identifier: NCT04844450

## 1. Introduction

Metabolic dysfunction–associated steatotic liver disease (MASLD), formerly known as nonalcoholic fatty liver disease (NAFLD), represents one of the most pervasive chronic liver diseases globally [1]. This condition significantly impacts public health on an international scale. MASLD is characterized by hepatic steatosis and a combination of other metabolic risk factors and can progress to cirrhosis and hepatocellular carcinoma [2, 3]. Although previous studies have shown that beneficial lifestyle and exercise can slow the progression of fatty liver, they are incapable of reversing existing lesions [4]. Therefore, finding and developing pharmacologic interventions for MASLD is very attractive.

Genome-wide association studies (GWASs) are a method for identifying associations between genetic regions (genomes) and traits/diseases. Through GWAS analysis, the researchers found that genetic variants closely associated with MASLD were located near the patatin-like phospholipase domain-containing protein 3 (PNPLA3), TM6SF2, and HSD17B1 genes [4]. The results of the GWAS analysis suggest the involvement of these three genes in the development of MASLD and the potential as therapeutic targets for MASLD. Additionally, Liu et al. found that individuals carrying the PNPLA3 rs738409 C > G gene polymorphism (I148M) had a higher risk of developing MASLD and liver fibrosis [5]. Animal model studies have further demonstrated that variants of the PNPLA3 protein in the liver influence the equilibrium of hepatic lipid metabolism [6]. Such research implies that PNPLA3 might serve as a promising therapeutic target for impeding the progression of MASLD. Currently, ARO-PNPLA3 is a newly developed therapy for MASLD. ARO-PNPLA3 reduced liver PNPLA3 protein expression and decreases liver fat in MASLD patients [7]. In phase I clinical trial, ARO-PNPLA3 has shown exciting results and may bring a new and unique therapeutic approach to the treatment of MASLD. However, the restricted clinical trial data do not indicate safety in long-term therapy. Consequently, considering the correlation between MASLD onset and metabolic function, it is essential to further examine the impact of PNPLA3 inhibition on other metabolic-related diseases.

Gout is a metabolic disease caused by abnormal metabolism of purines, resulting in increased synthesis of blood urate or decreased excretion of urate [8]. Multiple cross-sectional investigations and meta-analyses have demonstrated the association between urate and MASLD [9–12]. Nevertheless, ARO-PNPLA3, a prospective therapeutic agent for MASLD, is still unknown for the effect of PNPLA3 inhibition on urate and gout.

Mendelian randomization (MR) has become a widely adopted analytical method to assess the relationship between genetically predicted exposure and outcomes [13]. In this study, we proposed a two-sample MR approach to explore the association between PNPLA3 inhibition and gout. Furthermore, given the strong correlation between lipids and MASLD, we employed a two-step MR to assess the potential role of plasma lipids in the relationship between PNPLA3 and gout. The objective of this research is to offer insights for subsequent clinical trials or future clinical applications of PNPLA3 inhibitors.

## 2. Methods

### 2.1. Study Design

**Figure 1:**
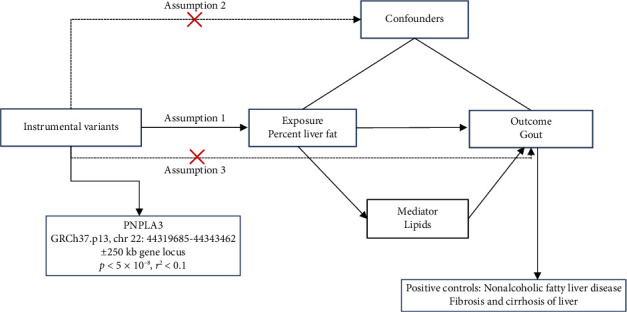
MR study design.

This study was designed according to the Strengthening the Reporting of Observational Studies in Epidemiology using Mendelian Randomization (STROBE-MR). The design for MR was required to meet three assumptions: (1) a strong correlation exists between the instrumental variable representing the genetic variant and the exposure; (2) the genetic variant is not associated with confounders; and (3) the genetic variant influences the outcome exclusively through the exposure (Figure 1). Since this investigation solely utilized data from publicly accessible GWAS, it was not mandatory to seek additional ethical review from the institutional review board.

### 2.2. Selection of Instrumental Variables

The GWAS data for percent liver fat were derived from a real-world UK Biobank dataset encompassing 32,858 participants, in which liver fat was meticulously gauged and quantified by the researchers [14]. To symbolize the function of PNPLA3 inhibition, we employed the percentage of liver fat as a biomarker. We first extracted all single-nucleotide polymorphisms (SNPs) located ±250 kb around the PNPLA3 gene (GRCh37/hg19, chr22: 44,316,685–44,343,462) that were genome-wide significant for percentage of liver fat (*P* < 5 × 10^−8^). Then, we pruned these SNPs to remove linkage disequilibrium (*r*^2^ < 0.1) We further computed the F-statistics of SNPs to eliminate weak instrumental variables (F-statistics < 10). Based on the MR hypothesis, a total of 8 SNPs were identified (Table s1). The F-statistics of the selected IVs were all above 10, suggesting that the conclusions are unlikely to be influenced by weak IVs.

### 2.3. Genetic Instruments for Mediators and Outcomes

GWAS data related to gout outcomes were obtained from FINNGEN and encompassed gout (3576 instances and 147,221 counterparts), idiopathic gout (819 instances and 215,216 counterparts), and gout due to impaired renal function (92 cases and 215,216 controls) [15]. Urate data were derived from a GWAS analysis of 110,347 participants of European ancestry by the Global Urate Genetics Consortium (GUGC) [16]. Data on NAFLD (894 cases and 217,898 controls) and liver fibrosis and cirrhosis (811 cases and 213,592 controls) in the positive controls were also obtained from FINNGEN. Given the correlation between plasma lipid levels and the occurrence of MASLD, we also examined the potential mediating influence of several lipids, including triglyceride (TG), total cholesterol (TC), LDL cholesterol (LDL-C), apolipoprotein A-I (ApoA1), and apolipoprotein B (ApoB). The data for TC were sourced from the Global Lipids Genetics Consortium (GLGC) [17], while the data for the remaining lipids were derived from a study conducted by the UK Biobank [18].

### 2.4. Statistical Analysis

The primary analytical approach employed was inverse variance weighting (IVW). This was supplemented with MR Egger, weighted median, simple mode, and weighted mode techniques [19]. The pleiotropy of IVs was evaluated using the MR-PRESSO global test and MR-Egger regression, while Cochran's *Q* test was utilized to assess heterogeneity. Additionally, leave-one-out analyses were conducted to ascertain the impact of individual SNPs on MR estimates. We conducted a two-step MR analysis to investigate the role of lipid traits in mediating the relationship between PNPLA3 inhibition and gout. We first estimated the effect of PNPLA3 inhibition on lipid (*β*1). Subsequently, we assessed the impact of lipids on gout (*β*2). The proportion of each lipid mediating the association between PNPLA3 inhibition and gout was determined by multiplying *β*1 and *β*2 and then dividing by the total effect of PNPLA3 on gout. All analyses were executed using R software (Version 4.1.3), with MR analyses and sensitivity analyses performed utilizing the TwoSampleMR R package (Version 0.5.6) and the MRPRESSO package (Version 1.0).

## 3. Results

### 3.1. MR Analysis to Estimate the Effects of PNPLA3 Inhibition on Positive Controls

**Figure 2:**
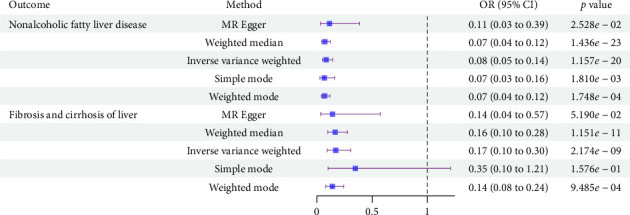
Forest plot for estimating the effect of PNPLA3 inhibition on liver diseases. OR, odds ratio; CI, confidence interval.

As shown in Figure 2, PNPLA3 inhibition was associated with a reduced risk of NAFLD (OR: 0.08, 95% CI: 0.05 to 0.14, *p*=1.157 × 10^−20^) and fibrosis and cirrhosis of the liver (OR: 0.17, 95% CI: 0.10 to 0.30, *p*=2.174 × 10^−9^) (Figure s1). Sensitivity analyses did not show heterogeneity and pleiotropy (Table s2). In addition, MR-PRESSO analysis did not identify any potential pleiotropic SNP associated with liver disease outcomes (Table s2). Leave-one-out analysis indicated that the association between PNPLA3 inhibition and liver diseases was unlikely to be significantly influenced by any single SNP (Figure s2).

### 3.2. MR Analysis to Estimate the Effects of PNPLA3 Inhibition on Gout and Urate

**Figure 3:**
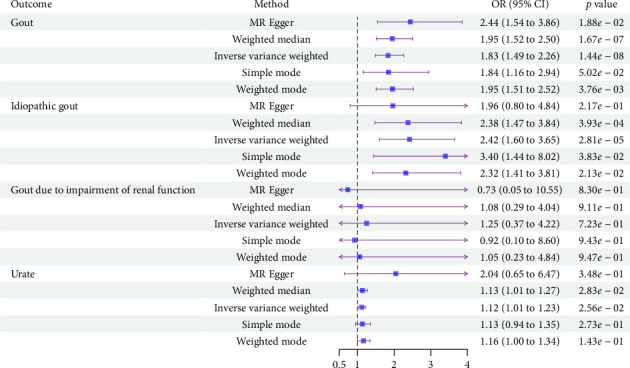
Forest plot for estimating the effect of PNPLA3 inhibition on gout and urate. OR, odds ratio; CI, confidence interval.

As shown in Figure 3, genetically predicted PNPLA3 inhibition resulted in an increased risk of gout (OR: 1.83, 95% CI: 1.49 to 2.26, *p*=1.44 × 10^−8^) and idiopathic gout (OR: 2.42, 95% CI: 1.60 to 3.65, *p*=2.81 × 10^−5^) (Figure s3), but not for gout due to impaired renal function (OR: 1.25, 95% CI: 0.37 to 4.22, *p*=7.23 × 10^−1^). In addition, genetically predicted PNPLA3 inhibition also elevated urate levels (OR: 1.12, 95% CI: 1.01 to 1.23, *p*=2.56 × 10−2). Sensitivity analyses did not show heterogeneity and pleiotropy (Table s3). MR-PRESSO analysis did not identify any potential pleiotropic SNP associated with gout and urate (Table s3). Leave-one-out analysis showed that the association between PNPLA3 inhibition and gout and urate was not significantly affected by any single SNP (Figure s4).

### 3.3. Mediation Analysis

**Figure 4:**
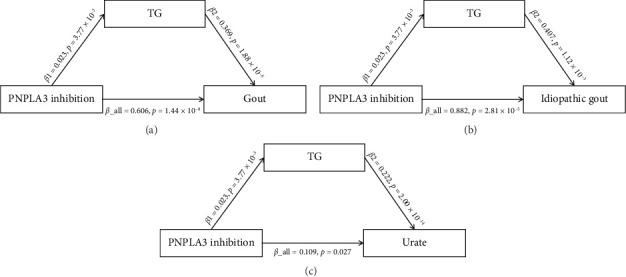
Associations between PNPLA3 inhibition and gout mediated by blood TG. TG, triglyceride.

For lipids, genetically predicted PNPLA3 inhibition elevated TG (β: 0.023, se: 0.008, *p*=3.77 × 10^−3^), LDL-C (*β*: 0.026, se: 0.008, *p*=1.51 × 10^−3^), and ApoA1 (*β*: 0.091, se: 0.009, *p*=4.60 × 10^−26^) levels but was not significantly associated with TC or ApoB (Table s4). Sensitivity analysis showed no pleiotropy or heterogeneity (Table s5). Subsequently, we confirmed whether TG, LDL-C, and ApoA1 played roles in the genetically predicted link between PNPLA3 inhibition and gout. The findings indicate that LDL-C and ApoA1 are improbable mediators of the genetically predicted association between PNPLA3 inhibition and gout (Table S6). However, blood TG may mediate the genetically predicted association between PNPLA3 inhibition and gout and urate (Figure 4 and Table s7).

## 4. Discussion

As far as we know, this is the first study on the association between PNPLA3 inhibition and gout. In this study, we simulated the impact of PNPLA3 inhibition by choosing SNPs adjacent to the PNPLA3 gene as instrumental variables, utilizing liver fat percentage as a biomarker. The selected instrumental variables notably diminished the risk of NAFLD and liver fibrosis, indicating that these variables accurately represent the effects of PNPLA3 inhibition. Moreover, we discovered that genetically predicted PNPLA3 inhibition could elevate the risk of idiopathic gout, gout, and high urate levels. Further analysis revealed that PNPLA3 inhibition might elevate TG, LDL-C, and ApoA1 levels, and TG may partially mediate the association between PNPLA3 inhibition and gout.

MASLD, previously known as NAFLD, has a relatively complex pathogenesis [20]. Due to the increasing incidence of MASLD, the search for therapeutic MASLD targets has attracted extensive attention from researchers. With the progress of GWASs in recent years, researchers have found a strong link between PNPLA3 and MASLD [21, 22]. The PNPLA3 protein is predominantly localized on the cytosolic lipid droplets (LDs) of liver and adipose tissues. It is known to possess TG hydrolase activity, which facilitates TG metabolism [23]. The PNPLA3 I148M mutation impairs TG mobilization within the LP by preventing ubiquitination and reducing degradation, which consequently leads to TG accumulation [6]. Researchers have also discovered that treating mice with antisense oligonucleotides targeting the PNPLA3 gene suppressed the expression of the PNPLA3 I148M variant, slowed the progression of NAFLD, and improved liver fibrosis [24]. Inhibiting the expression of the PNPLA3 protein may serve as an effective therapeutic approach for MASLD patients harboring the I148M mutation. In addition to its association with MASLD, some prospective studies have also suggested that PNPLA3 variants are associated with chronic kidney disease [25], and patients carrying PNPLA3 variants have a higher risk of early glomerular and tubular damage [26]. Kidney disease may lead to impaired uric acid excretion, which in turn may affect the occurrence of gout.

ARO-PNPLA3, a therapeutic agent that targets PNPLA3 mRNA to reduce its expression in hepatocytes, has shown promising results in reducing liver fat content without causing serious adverse events in a phase I clinical trial [7]. However, the complete function of PNPLA3 is yet to be fully understood, and its role in the progression of MASLD requires further investigation. Consequently, it is imperative to continue evaluating the viability and safety of PNPLA3 as a potential therapeutic target for MASLD. Gout is a metabolic disease associated with urate levels. Persistently high levels of urate are a major risk factor for gout. In addition, impaired renal function, resulting in impaired urate excretion, can also lead to gout. Urate levels are also closely related to lipid metabolism. A study by Wang et al. demonstrated a correlation between elevated urate levels and increased TG and visceral fat content [27]. Additionally, another prospective study indicated that high levels of TG and LDL-C are associated with an increased risk of hyperuricemia [28]. In addition, the MR study by Yu et al. also showed that TG levels were also positively correlated with serum urate [29], which is consistent with our results. In this MR study, genetically predicted PNPLA3 inhibition was associated with an increased risk of elevated urate levels and gout. However, there was no association between PNPLA3 inhibition and gout due to impaired renal function. These findings suggest that the mechanism by which PNPLA3 inhibition might increase the risk of gout is unlikely to involve direct effects on kidney function. Further, mediation analyses revealed that elevated TG levels were associated with an increased risk of high urate level and gout. Additionally, these analyses indicated that blood TG levels may act as a mediator in the relationship between PNPLA3 inhibition and the increased risk of gout. Although there was no direct evidence to indicate the molecular mechanism of PNPLA3 inhibition in gout, based on previous studies of PNPLA3 and our MR results, we speculated that PNPLA3 inhibition leads to LD accumulation, thereby disrupting LD remodeling and triggering mitochondrial dysfunction, excessive ROS, and lipid peroxidation products [6, 30, 31]. The excessive ROS activated the NLRP3 inflammasome, promoting the release of proinflammatory factors such as IL-1β, which further damages renal tubular epithelial cells, inhibited the function of uric acid transporters such as ABCG2 and URAT1, and caused impaired uric acid excretion, ultimately leading to the onset of gout. Therefore, it is essential to closely monitor whether ARO-PNPLA3 administration results in alterations in blood TG levels and increased urate during subsequent clinical trials. Furthermore, additional research is needed to determine if the inhibition of PNPLA3 due to prolonged pharmacological use elevates the risk of gout.

There are several limitations to our study that should be considered. First, the data selected for analysis were derived exclusively from European populations and did not include individuals from other ethnic or racial backgrounds. Second, the genetic instrument used in this study represents the lifetime effect of PNPLA3 activity rather than the short-term inhibitory effects of a pharmacological agent. These limitations underscore the need for further research to validate and extend these findings to more diverse populations and to specifically assess the short-term effects of PNPLA3 inhibition as would occur with drug treatment.

## 5. Conclusion

In conclusion, this study found that PNPLA3 inhibition increased the risk of high uric acid levels and gout. Additionally, PNPLA3 inhibition was associated with elevated TG levels, which partially mediated the relationship between PNPLA3 inhibition and an increased risk of gout. These findings provide important insights that will aid in the design and assessment of future clinical trials involving PNPLA3 inhibitors.

## Data Availability

The data that support the findings of this study are available from the corresponding author upon reasonable request.

## Supporting Information

Figure s1. Scatter plots for the effect of PNPLA3 inhibition on liver diseases. A: nonalcoholic fatty liver disease; B: fibrosis and cirrhosis of liver.

Figure s2. Leave-one-out analysis of PNPLA3 inhibition on liver diseases. A, nonalcoholic fatty liver disease; B, fibrosis and cirrhosis of liver.

Figure s3. Scatter plots for the effect of PNPLA3 inhibition on gout and urate. A, gout; B, idiopathic gout; C, urate.

Figure s4. Leave-one-out analysis of PNPLA3 inhibition on gout and urate. A, gout; B, idiopathic gout; C, urate.

Table s1. Information for instrumental variables to represent PNPLA3 inhibition.

Table s2. Sensitivity analysis of PNPLA3 inhibition on liver diseases.

Table s3. Sensitivity analysis of PNPLA3 inhibition on gout and urate.

Table s4. The effect of PNPLA3 inhibition on blood lipids.

Table s5. Sensitivity analysis of PNPLA3 inhibition on blood lipids.

Table s6. The effect of blood lipids on gout.

Table s7. Associations between PNPLA3 inhibition and gout mediated by blood TG.
