# Supplementary material for: Association Between PNPLA3 Inhibition and Gout: A Drug Target Mendelian Randomization Study
Source: Int J Endocrinol. 2025 Aug 20;2025:6664846. doi: 10.1155/ije/6664846 (PMC12390521; doi:10.1155/ije/6664846)
Supplement: Supporting Information — Additional supporting information can be found online in the Supporting Information section. [file 6664846.f1.docx]

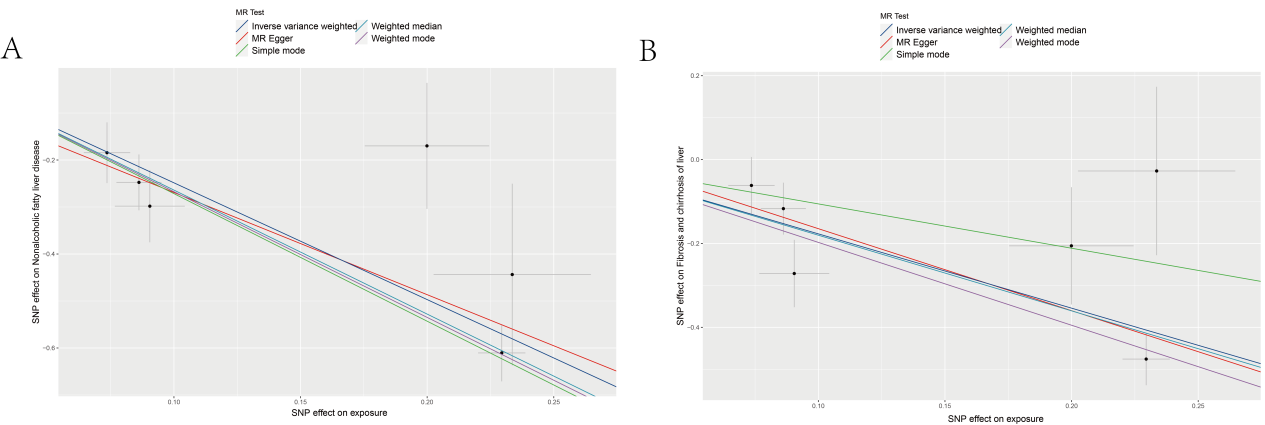


**Figure s1.** Scatter plots for the effect of PNPLA3 inhibition on liver diseases. A: Nonalcoholic fatty liver disease; B: Fibrosis and chirrhosis of liver.


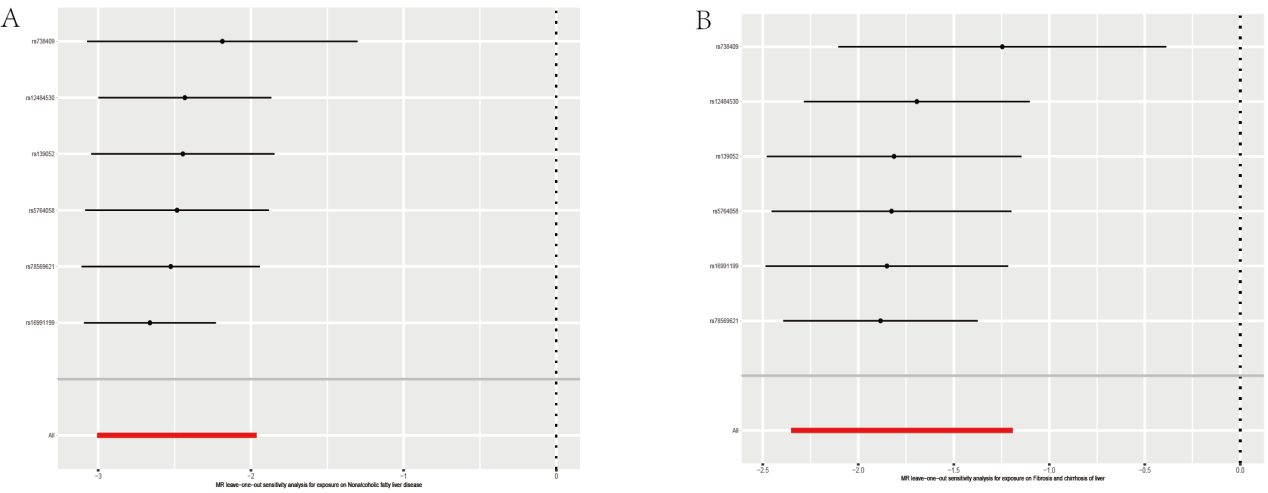


**Figure s2.** Leave-one-out analysis of PNPLA3 inhibition on liver diseases. A, Nonalcoholic fatty liver disease; B, Fibrosis and chirrhosis of liver.


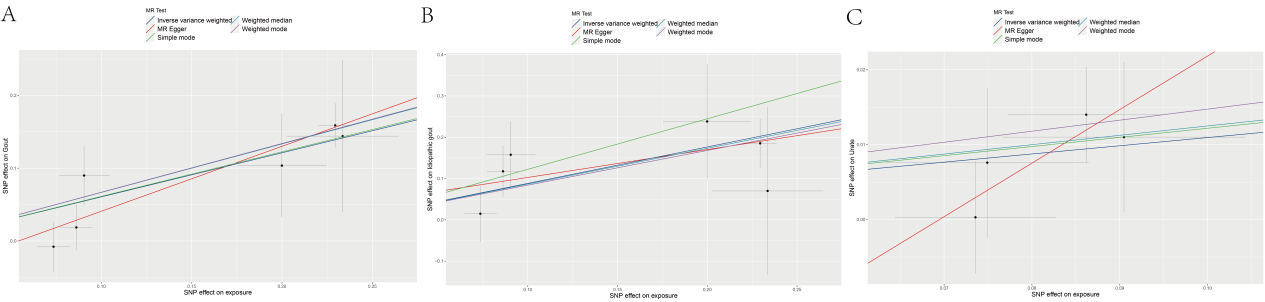


**Figure s3**. Scatter plots for the effect of PNPLA3 inhibition on gout and urate. A, Gout; B, Idiopathic gout; C, Urate.


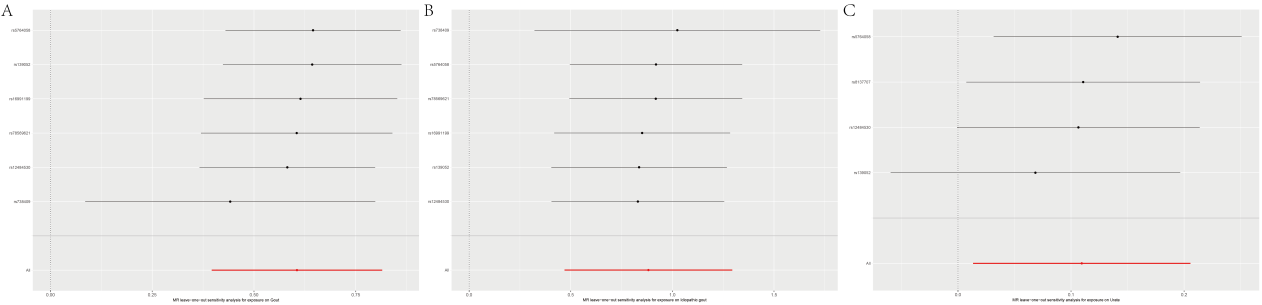


**Figure s4**. Leave-one-out analysis of PNPLA3 inhibition on gout and urate. A, Gout; B, Idiopathic gout; C, Urate.

| SNP | Effect_allele | Other_allele | Beta | SE | P-value | EAF | F statistic |
| --- | --- | --- | --- | --- | --- | --- | --- |
| rs5764058 | G | A | 0.074 | 0.009 | 9.90E-16 | 0.755 | 64.446 |
| rs78569621 | T | C | 0.234 | 0.031 | 5.40E-14 | 0.015 | 56.584 |
| rs12484530 | A | G | 0.090 | 0.014 | 5.70E-11 | 0.084 | 42.902 |
| rs148122504 | G | A | 0.246 | 0.023 | 1.00E-26 | 0.031 | 114.423 |
| rs8137707 | T | C | 0.075 | 0.012 | 9.30E-11 | 0.127 | 41.967 |
| rs738409 | G | C | 0.229 | 0.009 | 1.50E-133 | 0.214 | 604.826 |
| rs16991199 | G | T | 0.200 | 0.025 | 3.90E-16 | 0.025 | 66.281 |
| rs139052 | A | C | 0.086 | 0.009 | 3.20E-22 | 0.747 | 93.941 |

**Table s1**. Information for instrumental variables to represent PNPLA3 inhibition.

SNP: single nucleotide polymorphism; EAF: effect allele frequency.

**Table s2**. Sensitivity analysis of PNPLA3 inhibition on liver diseases.

| **Outcome** | **Heterogeneity** | | **Pleiotropy** | |  |
| --- | --- | --- | --- | --- | --- |
|  | **IVW Q (P-value)** | **MR-Egger Q (P-value)** | **MR-Egger’s intercept** | **SE (P-value)** | **MR-PRESSO RSSobs (P-value)** |
| Nonalcoholic fatty liver disease | 8.125 (0.149) | 7.536(0.110) | -0.052 | 0.092 (0.606) | 12.521 (0.335) |
| Fibrosis and chirrhosis of liver | 9.356 (0.096) | 9.163 (0.057) | 0.031 | 0.106 (0.786) | 18.618 (0.243) |

**Table s3**. Sensitivity analysis of PNPLA3 inhibition on gout and urate.

| **Outcome** | **Heterogeneity** | | **Pleiotropy** | |  |
| --- | --- | --- | --- | --- | --- |
|  | **IVW Q (P-value)** | **MR-Egger Q (P-value)** | **MR-Egger’s intercept** | **SE (P-value)** | **MR-PRESSO RSSobs (P-value)** |
| Gout | 4.755 (0.447) | 2.848 (0.584) | -0.048 | 0.035 (0.240) | 8.530 (0.483) |
| Idiopathic gout | 2.672 (0.750) | 2.416 (0.660) | 0.035 | 0.069 (0.639) | 3.654 (0.813) |
| Gout due to impairment of renal function | 1.006 (0.962) | 0.812 (0.937) | 0.090 | 0.205 (0.683) | 1.407 (0.944) |
| Urate | 1.593 (0.661) | 0.525 (0.769) | -0.050 | 0.048 (0.410) | 3.435 ( 0.654) |

**Table s4**. the effect of PNPLA3 inhibition on blood lipids.

| **Outcome** | **Method** | **Beta** | **SE** | **P-value** |
| --- | --- | --- | --- | --- |
| TG | Inverse variance weighted | 0.023 | 0.008 | 3.77E-03^*^ |
| TC | Inverse variance weighted | 0.022 | 0.047 | 6.33E-01 |
| LDL-C | Inverse variance weighted | 0.026 | 0.008 | 1.51E-03^*^ |
| ApoA1 | Inverse variance weighted | 0.091 | 0.009 | 4.60E-26^*^ |
| ApoB | Inverse variance weighted | -0.016 | 0.008 | 5.80E-02 |

TG, triglyceride; TC, total cholesterol; LDL-C, low-density lipoprotein cholesterol; ApoA1, apolipoprotein A-Ⅰ; ApoB, apolipoprotein B. * P < 0.05.

**Table s5**. Sensitivity analysis of PNPLA3 inhibition on blood lipids.

| **Exposure** | **Outcome** | **Heterogeneity** | | **Pleiotropy** | |  |
| --- | --- | --- | --- | --- | --- | --- |
|  |  | **IVW Q (P-value)** | **MR-Egger Q (P-value)** | **MR-Egger’s intercept** | **SE (P-value)** | **MR-PRESSO RSSobs (P-value)** |
| PNPLA3 inhibition | TG | 6.495 (0.483) | 4.627 (0.593) | -0.003 | 0.002 (0.221) | 11.441 (0.433) |
|  | LDL-C | 6.645 (0.467) | 5.774 (0.450) | -0.002 | 0.002 (0.387) | 8.972 (0.541) |
|  | ApoA1 | 8.474 (0.293) | 8.387 (0.211) | 0.001 | 0.003 (0.812) | 12.197 (0.387) |

TG, triglyceride; LDL-C, low-density lipoprotein cholesterol; ApoA1, apolipoprotein A-Ⅰ.

**Table s6**. the effect of blood lipids on gout.

| **Exposure** | **Outcome** | **Method** | **Beta** | **SE** | **P-value** |
| --- | --- | --- | --- | --- | --- |
| LDL-C | Gout | Inverse variance weighted | -0.058 | 0.091 | 0.521 |
|  | Idiopathic gout | Inverse variance weighted | -0.255 | 0.141 | 0.070 |
|  | Urate | Inverse variance weighted | -0.050 | 0.067 | 0.454 |
| ApoA1 | Gout | Inverse variance weighted | -0.102 | 0.079 | 0.197 |
|  | Idiopathic gout | Inverse variance weighted | 0.063 | 0.133 | 0.639 |
|  | Urate | Inverse variance weighted | -0.065 | 0.040 | 0.099 |

LDL-C, low-density lipoprotein cholesterol; ApoA1, apolipoprotein A-Ⅰ.

**Table s7**. Associations between PNPLA3 inhibition and gout mediated by blood TG.

| **Outcome** | **Mediation** | **Total effect(Beta)** | **β1 (Beta)** | **β2 (Beta)** | **Indirect effect (Beta)** | **Mediation effect/ Total effect** |
| --- | --- | --- | --- | --- | --- | --- |
|  |  |  |  |  |  |  |
| Gout | Triglyceride | 0.606 | 0.023 | 0.369 | 0.008 | 0.006 |
| Idiopathic gout | Triglyceride | 0.882 | 0.023 | 0.407 | 0.009 | 0.005 |
| Urate | Triglyceride | 0.109 | 0.023 | 0.222 | 0.005 | 0.017 |
